# The Expression Patterns and Associated Clinical Parameters of Human Endogenous Retrovirus-H Long Terminal Repeat-Associating Protein 2 and Transmembrane and Immunoglobulin Domain Containing 2 in Oral Squamous Cell Carcinoma

**DOI:** 10.1155/2019/5421985

**Published:** 2019-04-07

**Authors:** Yao Xiao, Hao Li, Lei-Lei Yang, Liang Mao, Cong-Cong Wu, Wen-Feng Zhang, Zhi-Jun Sun

**Affiliations:** ^1^The State Key Laboratory Breeding Base of Basic Science of Stomatology (Hubei-MOST) & Key Laboratory of Oral Biomedicine Ministry of Education, School & Hospital of Stomatology, Wuhan University, Wuhan, China; ^2^Department of Oral Maxillofacial-Head Neck Oncology, School & Hospital of Stomatology, Wuhan University, Wuhan, China

## Abstract

Human endogenous retrovirus-H long terminal repeat-associating protein 2 (HHLA2) and transmembrane and immunoglobulin domain containing 2 (TMIGD2) are new immune checkpoint molecules of the B7:CD28 family; however, little research has been performed on these immune checkpoint molecules. In this study, we used oral squamous cells carcinoma (OSCC) tissue microarrays and immunohistochemistry methods to investigate the expression patterns of HHLA2 and TMIGD2 in OSCC. After comparing the HHLA2 and TMIGD2 expression levels in OSCC, dysplasia, and mucosa, we found increased HHLA2 expression in OSCC and dysplasia, while the TMIGD2 expression was decreased in OSCC and dysplasia. Using the Kaplan-Meier method and log-rank test, we found that higher HHLA2 or TMIGD2 expression levels in OSCC indicate poor prognosis. Furthermore, two-tailed Pearson's statistical analysis revealed that the HHLA2 expression levels in OSCC, dysplasia, and mucosa were positively correlated with the T cell immunoglobulin and mucin-domain containing-3 (TIM3), lymphocyte-activation gene 3 (LAG3), B7 homolog 3 protein (B7-H3), B7 homolog 4 protein (B7H4), and V-domain Ig suppressor of T cell activation (VISTA) levels, while the TMIGD2 expression levels in OSCC, dysplasia, and mucosa were inversely correlated with the TIM3, LAG3, and B7H3 levels. Our current study demonstrates that HHLA2 may serve as an immune target for OSCC therapy and that the TMIGD2 expression level in OSCC could forecast patient prognosis.

## 1. Introduction

B7:CD28 family pathways have key roles in orchestrating T cell activation and tolerance and are promising therapeutic targets [1]. Interruption of the negative signals in B7:CD28 family pathways, which occurs after application of specific antibodies to block immune checkpoint molecules, can improve antitumor immunity and mediate stable cancer regression [2]. HHLA2 (B7H7/B7H5) and TMIGD2 (IGPR-1/CD28H) are new members of the B7:CD28 immune checkpoint family [3]. HHLA2 shares significant homology with the B7 family [4] and has been demonstrated to have both stimulatory and inhibitory properties on T cells [5]. TMIGD2 is a HHLA2 receptor [5], and it is expressed by all naive T cells, the majority of natural killer (NK) cells and half of the memory T cells, although it is not expressed by T regulatory cells or B cells [5]; moreover, TMIGD2 expression is lost in activated T cells [6]. The HHLA2/TMIGD2 interaction selectively costimulates human T cell growth and cytokine production via an AKT-dependent signaling cascade [6]. Moreover, TMIGD2 could also play an important role in cell-cell interactions, cell migration, and angiogenesis [7]. However, HHLA2 and TMIGD2 are both absent in mouse and rat [7, 8].

OSCC is a malignancy that occurs in the oral cavity and oropharyngeal region, and it has many risk factors [6, 9, 10]. One risk factor, the immunosuppressive status of OSCC [11], has drawn more attention, and the overexpression of immune checkpoint molecules in OSCC, as well as the effectiveness of immune checkpoint blockade therapy, has also been demonstrated [12]. OSCC is a prevalent cancer worldwide [13], and the 5-year survival rate is still very low, despite the introduction of radiation therapy and TPF (inductive taxol, platinum, and 5-fluorouracil) chemotherapy [14] in the treatment of OSCC. The limitations of radiotherapy and chemotherapy and the success of immunotherapy remind us that we should put more effort into OSCC immunotherapy research. New immune checkpoint molecules and the corresponding blockade therapeutic approaches should be explored. Currently, there is still little research about the roles of HHLA2 and TMIGD2 in OSCC.

In this study, we explored the expression patterns and associated clinical parameters of HHLA2 and TMIGD2 by immunohistochemistry methods on OSCC tissue microarrays, and we analyzed the quantified data of HHLA2 and TMIGD2 staining. Moreover, to explore the relationships between HHLA2 and TMIGD2 with other immune checkpoint molecules, we evaluated the correlations between HHLA2 and TMIGD2 expressions with TIM3, LAG3, B7H3, B7H4, and VISTA expression.

## 2. Materials and Methods

Detailed information is presented in the supplementary information.

### 2.1. Patient Samples and Tissue Microarrays

The samples used in this study were obtained from patients who received treatment at the School and Hospital of Stomatology, Wuhan University, from 2008 to 2017. Three sets of tissue microarrays (T12-412-TMA2, T15-411, and T17-490) were constructed.

### 2.2. Immunohistochemistry

The paraffin-embedded samples were cut into 4 *μ*m thick slices, incubated at 4°C overnight in primary antibodies against HHLA2 (Abcam, Cambridge, UK), TMIGD2 (Abcam), TIM3 (Cell Signaling Technology, Danvers, MA, USA), LAG3 (Cell Signaling Technology), B7H3 (Cell Signaling Technology), VISTA (Cell Signaling Technology), and B7H4 (Cell Signaling Technology). The signals were visualized by staining with diaminobenzidine and counterstaining with hematoxylin.

### 2.3. Scoring and Statistical Analysis

All of the sections were scanned with an Aperio ScanScope CS2 scanner (Vista, CA, USA), and the quantification was performed using Aperio Quantification software (version 9.1). The immunohistoscore data were analyzed in GraphPad Prism version 7.0 (GraphPad Software Inc., La Jolla, CA). The hierarchical analysis results were generated using Cluster 3.0 (with average linkage based on Pearson's correlation coefficient) [15] and were visualized using Java TreeView1.0.5 [16]. The statistical significance was defined as a *p* value < 0.05.

## 3. Results

### 3.1. HHLA2 Levels Are Increased in OSCC and Dysplasia, while TMIGD2 Levels Are Decreased in OSCC and Dysplasia, and Both Cases Indicate Poor Prognosis

**Figure 1:**
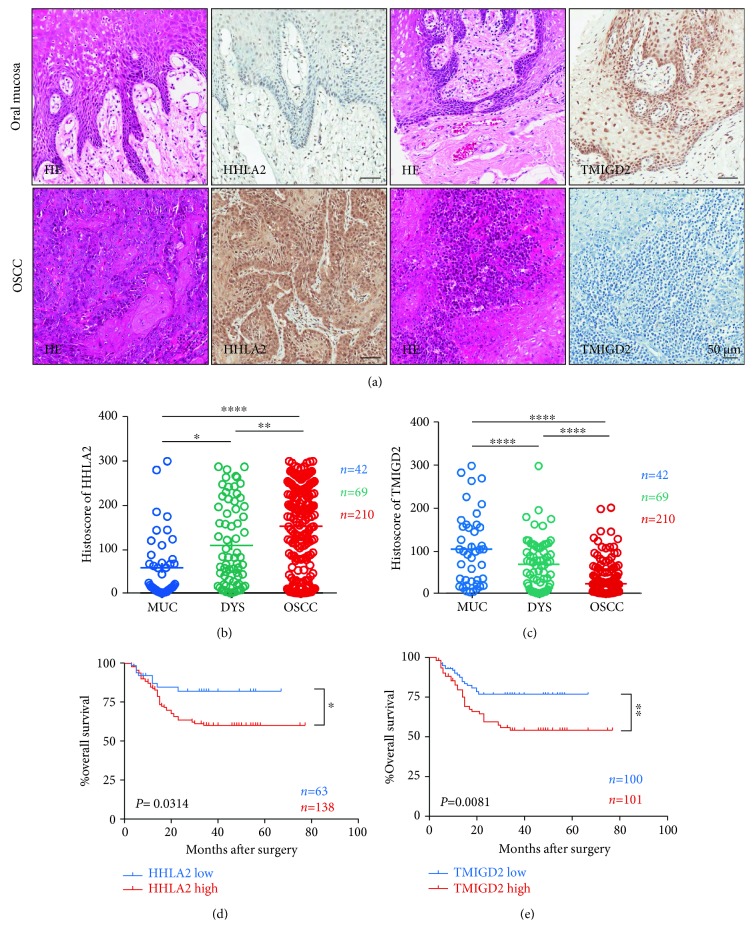
HHLA2 was overexpressed, while the TMIGD2 expression level was decreased in oral squamous cell carcinoma. (a) Hematoxylin and eosin (HE) staining and HHLA2 as well as TMIGD2 immunostaining of oral mucosa and oral squamous cell carcinoma. Scale bar: 50 *μ*m. (b) Continuously and significantly increased levels of HHLA2 expression from oral mucosa (MUC, *n* = 42) to dysplasia (DYS, *n* = 69) to OSCC (OSCC, *n* = 210). All data are presented as the mean ± standard error of the mean and analyzed by one-way ANOVA with Tukey's post hoc test. ^∗^ *p* < 0.05; ^∗∗^ *p* < 0.01; ^∗∗∗^ *p* < 0.001; ^∗∗∗∗^ *p* < 0.0001. (c) Continuously and significantly increased levels of TMIGD2 expression from oral mucosa (MUC, *n* = 42) to dysplasia (DYS, *n* = 69) to OSCC (OSCC, *n* = 210). (d) The overall survival rate of the group of patients with lower HHLA2 expression (*n* = 68) was significantly different from that of the group of patients with higher HHLA2 expression (*n* = 138, *p* = 0.0314, cutoff = 85.4). (e) The overall survival rate of the group of patients with lower TMIGD2 expression (*n* = 100) was significantly different from that of the group of patients with higher TMIGD2 expression (*n* = 101, *p* = 0.0081; the median expression of TMIGD2 was used as the cutoff).

To investigate the expression patterns of HHLA2 and TMIGD2 in OSCC, immunohistochemical staining was performed on tissue microarrays. We found that most of the OSCC tissue samples, and rarely normal mucosa tissue, were stained by anti-HHLA2 antibody; on the contrary, most of the normal mucosa tissue samples, and rarely the OSCC tissue samples, were stained with anti-TMIGD2 antibody (Figure 1(a)). The quantification and statistical analysis of the staining patterns of HHLA2 and TMIGD2 in the normal mucosa, dysplasia, and OSCC samples revealed that the HHLA2 expression level was continuously and significantly increased in the dysplasia and OSCC samples (Figure 1(b)), while the TMIGD2 expression level was continuously and significantly decreased in the stroma of the dysplasia samples and in the stroma of the OSCC samples (Figure 1(c)). Survival analysis was conducted among 201 patients with primary OSCC, and nine patients were excluded due to loss at follow-up. The use of the Kaplan-Meier method revealed that higher HHLA2 expression indicated significantly lower overall survival rates at the best cutoff [17] (*p* = 0.0314, cutoff = 85.4, Figure 1(d)) and that higher TMIGD2 expression indicated significantly lower overall survival rates at the median cutoff (*p* = 0.0081, Figure 1(e)). However, higher HHLA2 expression could not predict significantly lower overall survival rates at the median cutoff (*p* = 0.6547, Figure S2C). Moreover, multivariate analysis revealed that HHLA2 level could not be used as an independent prognostic indicator at the median cutoff (*p* = 0.713, Supplementary Table 1) or at the best cutoff (*p* = 0.087, Supplementary Table 2); but HHLA2 was marginally correlated with prognosis at the best cutoff (*p* = 0.087, Supplementary Table 2), and TMIGD2 could be used as an independent indicator for poor prognosis (*p* = 0.029, Table 1).

### 3.2. HHLA2 And TMIGD2 Expression Levels Are Independent of the Pathology Grade, Tumor Size, and Lymph Node Stage as well as the Lymph Node Metastatic Status

**Figure 2:**
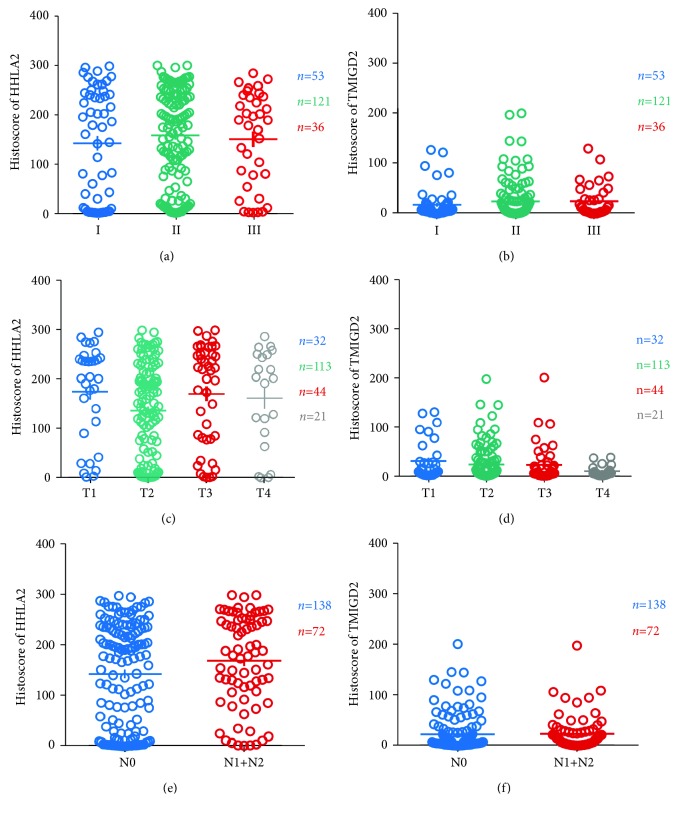
The HHLA2 and TMIGD2 expression levels were independent of OSCC pathology grade, tumor size, and lymph node metastasis. (a) No significant difference in HHLA2 expression was observed among the three pathology grades (I–III: I = 53, II = 121, and III = 36) of OSCC. (b) No significant difference in TMIGD2 expression was observed among the three pathology grades (I–III: I = 53, II = 121, and III = 36) of OSCC. (c) The expression levels of HHLA2 in the four stages (T1, T2, T3, and T4: T1 = 32, T2 = 113, T3 = 44, and T4 = 21) of tumor size showed no significant differences with each other. (d) The expression levels of TMIGD2 in the four stages (T1, T2, T3, and T4: T1 = 32, T2 = 113, T3 = 44, and T4 = 21) of tumor size showed no significant differences with each other. (e) There was no significant difference between the HHLA2 expression levels in OSCC cases with different lymph node metastasis status (negative: N0, *n* = 138; positive: N1+N2, *n* = 72). (f) There was no significant difference between the TMIGD2 expression levels in OSCC cases with different lymph node metastasis status (negative: N0, *n* = 138; positive: N1+N2, *n* = 72).

In our study, the expression levels of HHLA2 and TMIGD2 in different OSCC pathology grades, tumor sizes, and lymph node status were analyzed by the one-way analysis of variance method and *t*-test method. We found that the HHLA2 expression levels were not significantly changed in the different pathology grades (Figure 2(a)), and a similar result was also found for TMIGD2 (Figure 2(b)). Moreover, neither HHLA2 expression nor TMIGD2 expression was related to tumor size (Figures 2(c) and 2(d)). Furthermore, neither the HHLA2 expression level nor the TMIGD2 expression level showed significant differences between the tumors with lymph node metastasis and the tumors without lymph node metastasis (Figures 2(e) and 2(f)). The HHLA2 and TMIGD2 expression levels also showed no significant differences between the primary OSCC and corresponding metastatic lymph nodes (Figure S2. A, B).

### 3.3. TMIGD2 Expression Is Increased in Recurrent OSCC, but Neither HHLA2 Nor TMIGD2 Expression Is Associated with TPF Chemotherapy and Radiotherapy

**Figure 3:**
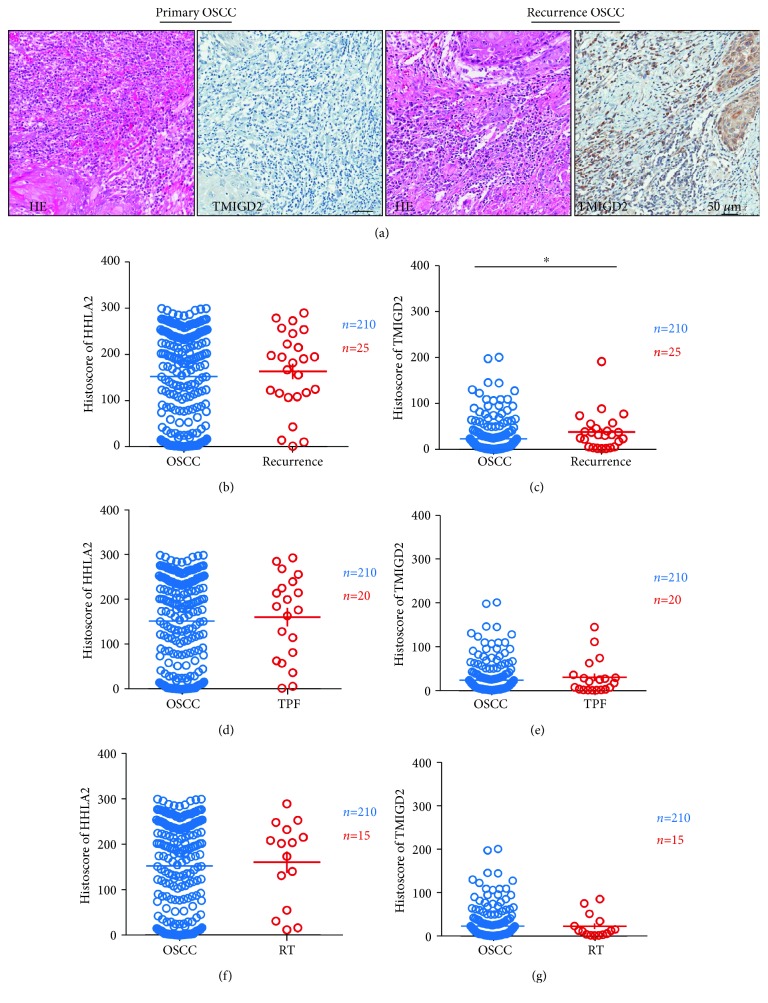
The TMIGD2 expression level was increased in recurrent OSCC. (a) Hematoxylin and eosin (HE) staining with TMIGD2 immunostaining of primary OSCC and recurrent OSCC. (b) There was no significant difference in the HHLA2 expression levels between primary OSCC and recurrent OSCC. (c) The TMIGD2 expression level was significantly increased in recurrent OSCC compared with primary OSCC. (d) There was no significant difference in the HHLA2 expression levels between primary OSCC and OSCC after TPF therapy. (e) There was no significant difference in the TMIGD2 expression levels between primary OSCC and OSCC after TPF therapy. (f) There was no significant change in the HHLA2 expression level in OSCC after radiotherapy compared with primary OSCC. (g) There was no significant change in the TMIGD2 expression level in OSCC after radiotherapy compared with primary OSCC.

In this study, we compared the expression levels of HHLA2 and TMIGD2 in primary OSCC and recurrent OSCC, OSCC after TPF therapy, and OSCC after radiotherapy. The TMIGD2 expression level was increased in recurrent OSCC (Figure 3(c)). However, there was no significant difference in the HHLA2 expression levels between primary OSCC with recurrence (Figure 3(b)), after TPF therapy (Figure 3(d)), and after radiotherapy (Figure 3(f)). Similarly, we also found that there was no significant association between the TMIGD2 expression level and TPF therapy (Figure 3(e)) or radiotherapy (Figure 3(g)).

### 3.4. HHLA2 And TMIGD2 Expression Levels Are Independent of Risk Factors Such as Smoking, Drinking, and HPV Infection

To explore the association between HHLA2 and TMIGD2 expression levels and risk factors, clinical parameters such as smoking, drinking, and HPV infection were included in this study. We divided the patients into the smoking and nonsmoking groups, drinking and nondrinking groups, and HPV infection and non-HPV infection groups and then compared the differences in the HHLA2 and TMIGD2 expression levels in the two parts of each group. We found that there was no significant association between the HHLA2 and TMIGD2 expression levels with smoking (Figure S1A, B), drinking (Figure S1C, D), or HPV infection (Figure S1E, F).

### 3.5. HHLA2 Expression Was Positively Correlated with the TIM3, LAG3, B7H3, B7H4, and VISTA Levels, while TMIGD2 Expression Was Negatively Correlated with the TIM3, LAG3, and B7H3 Levels

**Figure 4:**
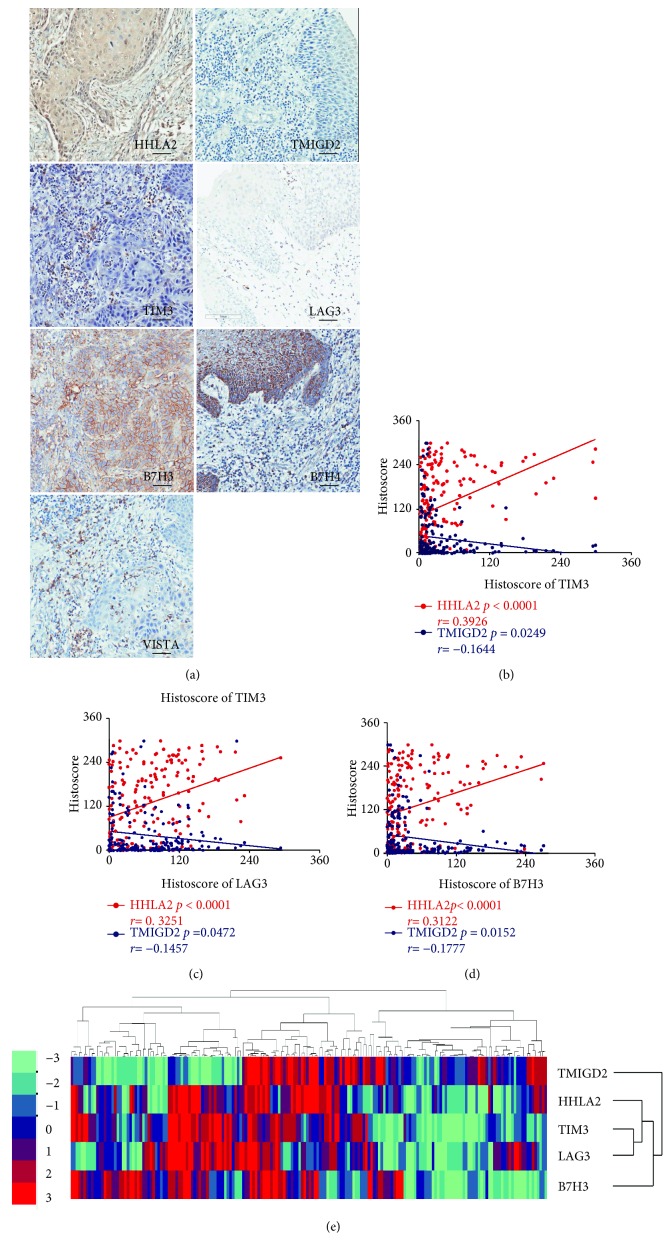


The continuously cut tissue microarray slices provided us with a good opportunity to explore the correlation between the HHLA2 and TMIGD2 expression patterns with another immune checkpoint molecules. After performing immunostaining of HHLA2, TMIGD2, TIM3, LAG3, B7H3, B7H4, and VISTA on the continuously cut tissue microarrays, we conducted two-tailed Pearson's tests to analyze the correlations between the HHLA2 and TMIGD2 expression patterns and the expression of TIM3, LAG3, B7H3, B7H4, and VISTA in the OSCC, dysplasia, and mucosa samples. Two tissue microarrays were applied to conduct this correlation analysis. We found that HHLA2 expression was positively correlated with the expression of TIM3 (*p* < 0.0001, *r* = 0.3926, Figure 4(b)), LAG3 (*p* < 0.0001, *r* = 0.3251, Figure 4(c)), B7H3 (*p* < 0.0001, *r* = 0.3122, Figure 4(d)), B7H4 (*p* < 0.0001, *r* = 0.3884, Figure S2D), and VISTA (*p* < 0.0001, *r* = 0.2858, Figure S2D). However, we also found that TMIGD2 expression was positively correlated with the expression of TIM3 (*p* = 0.0249, *r* = −0.1644, Figure 4(b)), LAG3 (*p* = 0.0472, *r* = −0.1457, Figure 4(c)), and B7H3 (*p* = 0.0152, *r* = −0.1777, Figure 4(d)), but TMIGD2 expression was not correlated with the expression of VISTA (*p* = 0.2664, *r* = −0.0819, Figure S2E) and B7H4 (*p* = 0.8134, *r* = 0.01742, Figure S2E).

## 4. Discussion

Immune checkpoint blockade therapy is a novel method of tumor therapy [18, 19]. Therefore, the exploration of new immune checkpoint factors may offer novel molecular targets in this tumor therapy method. In this study, we found that HHLA2 expression was increased in dysplasia and OSCC. Conversely, TMIGD2 expression was decreased in dysplasia and OSCC. Higher expression levels of both HHLA2 and TMIGD2 indicated poor prognosis, and TMIGD2 expression could be used as an independent prognosis indicator. Moreover, TMIGD2 expression was increased in recurrent OSCC. In addition, HHLA2 expression was positively correlated with TIM3, LAG3, B7H3, B7H4, and VISTA expression, while TMIGD2 expression was negatively correlated with TIM3, LAG3, and B7H3 expression.

Positive immune checkpoint molecules and negative immune checkpoint molecules are tightly controlled immune responses that allow effective clearance of invading pathogens or cancerous cells and yet maintain tolerance to self-factors [20]. However, imbalances of positive and negative immune checkpoint molecules in the tumor microenvironment lead to immune evasion by tumor cells [21, 22], and the expression levels of some negative immune checkpoint molecule are consistently elevated in tumors [12, 23–25]. HHLA2 is a member of the B7 family and has been shown to be broadly overexpressed in human cancers [26]. In this study, we also found that HHLA2 is overexpressed in OSCC. The expression of TMIGD2 in tumors has not been previously reported. In this study, we first report that TMIGD2 expression was decreased in OSCC. It may attribute to the complexity of the tumor microenvironment of OSCC, there is a dynamic interaction between the malignant cells of the OSCC and normal host immune cells [27], and activated T cells such as CD8 T cells are increased in OSCC compared with normal mucosa [12]; however, TMIGD2 is mainly expressed on naive T cell and its expression level is negatively correlated with T cell activation because T cell activation leads to loss of TMIGD2 expression [28]; based on this, we could observe that TMIGD2 decreased in OSCC compared with normal mucosa.

It has been shown that HHLA2 could play an immunosuppressive role in the tumor microenvironment [29] and that the expression of negative immune checkpoint molecules is always correlated with poor prognosis [12, 23–25]. Although TMIGD2 is a positive immune checkpoint molecule and transmits a positive stimulatory signal to naive T cells, its expression is negatively correlated with T cell activation because its expression is absent after T cell activation [5, 6]; activated T cells such as CD8 T cells are correlated with good prognosis [30]. Based on these observations, the HHLA2 or TMIGD2 expression levels may be correlated with poor prognosis in patients. However, the association between the HHLA2 and TMIGD2 expression levels and the overall survival rate of patients have not yet been reported. Our current study is the first to show that high levels of HHLA2 or TMIGD2 are both associated with poor prognosis. Moreover, Cox multivariate analysis confirmed that the TMIGD2 expression level could be used as an independent factor to indicate poor prognosis but HHLA2 was marginally correlated with prognosis at the best cutoff; further research will be done if follow-up time is long enough. Furthermore, we also found that TMIGD2 expression was increased in recurrent OSCC compared primary OSCC. TMIGD2 is an immune checkpoint molecule expressed in naive T cells and half of memory T cells [5]. The proportion of memory T cell has been reported to be increased in recurrent glioblastoma [31]. The high TMIGD2 expression level in recurrent OSCC suggested to us to explore the difference in the proportion of memory T cells in primary and recurrent OSCC, and further research will be conducted to investigate the correlation between TMIGD2 and recurrent tumors if a suitable sample or animal model is available.

TIM3, LAG3, B7H3, B7H4, and VISTA are negative immune checkpoint molecules, and their expression levels were increased in OSCC [23–25, 32, 33]. By taking advantage of the continuously cut slides, we explored the correlation between HHLA2 expression and the expression levels of TIM3, LAG3, B7H3, B7H4, and VISTA as well as the correlation between TMIGD2 expression and the expression levels of TIM3, LAG3, B7H3, B7H4, and VISTA. In these experiments, we found that HHLA2 expression was positively correlated with the TIM3, LAG3, B7H3, B7H4, and VISTA expression levels, while TMIGD2 was inversely correlated with the TIM3, LAG3, and B7H3 expression levels. Based on the positive correlation between HHLA2 and the levels of the negative immune checkpoint molecules as well as its high expression in OSCC, we hypothesized that HHLA2 may act as an immune suppressive molecule in OSCC. Moreover, combined immune checkpoint blockade therapy could be an available option for tumor immunotherapy [34], and the positive correlation between HHLA2 and negative immune checkpoint molecules suggests to us that we should explore a combination of HHLA2 with other immune checkpoint blockade therapies in future research.

## 5. Conclusion

HHLA2 expression was increased in OSCC, while TMIGD2 expression was decreased in OSCC, and elevated levels of HHLA2 and TMIGD2 were correlated with poor prognosis. In particular, TMIGD2 expression level could be used as an independent prognostic indicator. Moreover, more effort should be made to prove the immunosuppressive role of HHLA2 in OSCC and to investigate the potential of combination blockade therapy of HHLA2 and other immune checkpoint molecules.

## Figures and Tables

**Table 1:** TMIGD2 could offer as an independent factor to predict prognosis at median cutoff.

Parameters	HR (95% CI)	*p* value
Gender	0.900 (0.404-2.006)	0.796
Age	1.813 (0.983-3.341)	0.057
Pathological grade		
II vs. I	20.260 (2.733-150.209)	0.003^∗^
III vs. I	12.586 (1.599-99.072)	0.016^∗^
Tumor size		
T2 vs. T1	1.097 (0.440-2.734)	0.842
T3 vs. T1	1.817 (0.672-4.909)	0.239
T4 vs. T1	2.164 (0.716-6.542)	0.171
Node stage		
N1+N2 vs. N0	1.129 (0.628-2.029)	0.685
TMIGD2	1.924 (1.069-3.464)	0.029^∗^

Cox proportional hazards regression model. HR: hazard ratio; 95% CI: 95% confidence interval. ^∗^ *p* < 0.05.

## Data Availability

The HHLA2 and TMIGD2 expression data used to support the findings of this study have not been made available because these data are related to patient privacy.

## Supplementary Materials

Supplementary Materials Detailed materials and methods.
