# Supplementary material for: The Expression Patterns and Associated Clinical Parameters of Human Endogenous Retrovirus-H Long Terminal Repeat-Associating Protein 2 and Transmembrane and Immunoglobulin Domain Containing 2 in Oral Squamous Cell Carcinoma
Source: Dis Markers. 2019 Apr 7;2019:5421985. doi: 10.1155/2019/5421985 (PMC6476002; doi:10.1155/2019/5421985)
Supplement: Supplementary Materials — Detailed materials and methods. [file 5421985.f1.pdf]

The expression patterns and associated clinical parameters of human endogenous retrovirus-H long terminal repeat-associating protein 2 and transmembrane and immunoglobulin domain containing 2 in oral squamous cells carcinoma

Yao Xiao[1], Dr, Hao Li[1], Dr, Lei-Lei Yang[1], Dr, Liang Mao[1], Dr, Cong-Cong Wu[1], Dr, Wen-Feng Zhang[2], Dr, & Zhi-Jun Sun[1,2], DDS, PhD

[1]The State Key Laboratory Breeding Base of Basic Science of Stomatology (Hubei-MOST) & Key Laboratory of Oral Biomedicine Ministry of Education, School & Hospital of Stomatology, Wuhan University, Wuhan, China

[2]Department of Oral Maxillofacial-Head Neck Oncology, School & Hospital of Stomatology, Wuhan University, Wuhan, China

Corresponding authors:

Corresponding author:
Zhi-Jun Sun, DDS, PhD, School and Hospital of Stomatology, Wuhan University, Tel.: +86 27 8768 6108; Fax: +86 27 8787 3260; 237 Luoyu Road, Wuhan 430079, China.
Emails: sunzj@whu.edu.cn

Detailed materials and methods

Patients' samples and tissue microarrays

The samples involved in this study were obtained from patients who received treatment in School and Hospital of Stomatology, Wuhan University from 2008 to 2017. Until now, 42 normal oral mucosa lesions; 69 oral epithelial dysplasias; 210 primary OSCC lesions from patients without recurrent, pre-surgical inductive chemotherapy or pre-surgical radiotherapy; 20 OSCC lesions from patients who received pre-surgical TPF inductive chemotherapy (without pre-surgical radiotherapy); 15 OSCC

lesions from patients who received pre-surgical radiotherapy (without pre-surgical chemotherapy); 25 recurrent OSCC lesions; and 68 metastatic lymph node (35 metastatic lymph nodes with corresponding OSCC specimens and 33 metastatic lymph nodes without corresponding OSCC specimens) were obtained and diagnosed by two independent pathologist according to the guidelines of the International Union against Cancer (UICC2016). Each of samples was fixed by formalin for 48h after surgical resection, flowing water rinse for 24h and dehydrated by ethanol and N-butanol, and then trimmed as a 1.5mm core and embedded paraffin. Then three sets of tissue microarrays (T12-412-TMA2, T15-411, and T17-490) were constructed.

Immunochemistry

The paraffin-embedded samples were cut into slices of 4-μm thick, air-dried 2h at 60℃. Dimethylbenzene was applied to deparaffinize and then a descending series of alcohol solutions was used to hydrate. Following with antigen retrieval by 0.01M citric acid buffer solution (pH 6.0), hydrogen superoxide blocker and 10 % normal goat serum were applied sequentially to quench the endogenous peroxidase activity and block non-specific binding. After that, sections were incubated at 4℃ overnight within primary antibody HHLA2 (abcam, Cambridge, UK), TMIGD2 (abcam), TIM3 (Cell Signaling Technology, Danvers, MA, USA), LAG3 (Cell Signaling Technology), B7H3 (Cell Signaling Technology), VISTA (Cell Signaling Technology), B7H4 (Cell Signaling Technology), after that, all slides were incubated with secondary antibody and avidin-biotin-peroxidase reagent at 37℃ for 20min respectively. Diaminobenzidine as well as haematoxylin resulted in the visualization of the immunostaining and counterstaining. A positive control and negative isotype control were included in each experiment.

Scoring and statistical analysis

All the sections were scanned by Aperio ScanScope CS2 scanner (Vista, CA, USA), and qualification by Aperio Quantification software (Version 9.1). The histoscore of immunostaining was converted into Microsoft Excel and normalized between 0 and 300.

Multiple method was performed on Graph Pad Prism version 7.0 (GraphPad Software Inc, La Jolla, CA) to analysis the data. One-way analysis of variance was employed for comparing the different expression level of HHLA2 and TMIGD2 in three or more groups. Unpaired *t* tests and paired *t* tests were used for comparing the different expression level of HHLA2 and TMIGD2 in two groups. The Kaplan-Meier method and the log-rank test were applied to plot the Overall survival curves and assess whether the difference of overall survival rate in two groups was significant. Two tissue microarrays were applied to conduct correlation analysis (T12-412-TMA2, T15-411), all the samples exhibited a Gaussian distribution. Two-tailed Pearson's statistics was used to analysis the correlation between HHLA2 and TMIGD2 expression with TIM3, LAG3, B7H3, B7H4 and VISTA expression. The quantified results are presented as the mean ± standard error of the mean, and statistical significance was defined as a P-value < .05.

Hierarchical Clustering

After convert the expression level of HHLA2, TMIGD2, TIM3, LAG3 and B7H3 into scaled values between -3 and +3. The hierarchical analysis results were performed using Cluster 3.0 (with average linkage based on Pearson's correlation coefficient)[13] and were visualized using Java TreeView1.0.5[14]. The clustered data on behalf of the markers were displayed on the horizontal axis, and the representative tissue samples were arranged on the vertical axis. Closely related biomarkers were located tightly to each other.

Supplementary Figure

Figure.S1

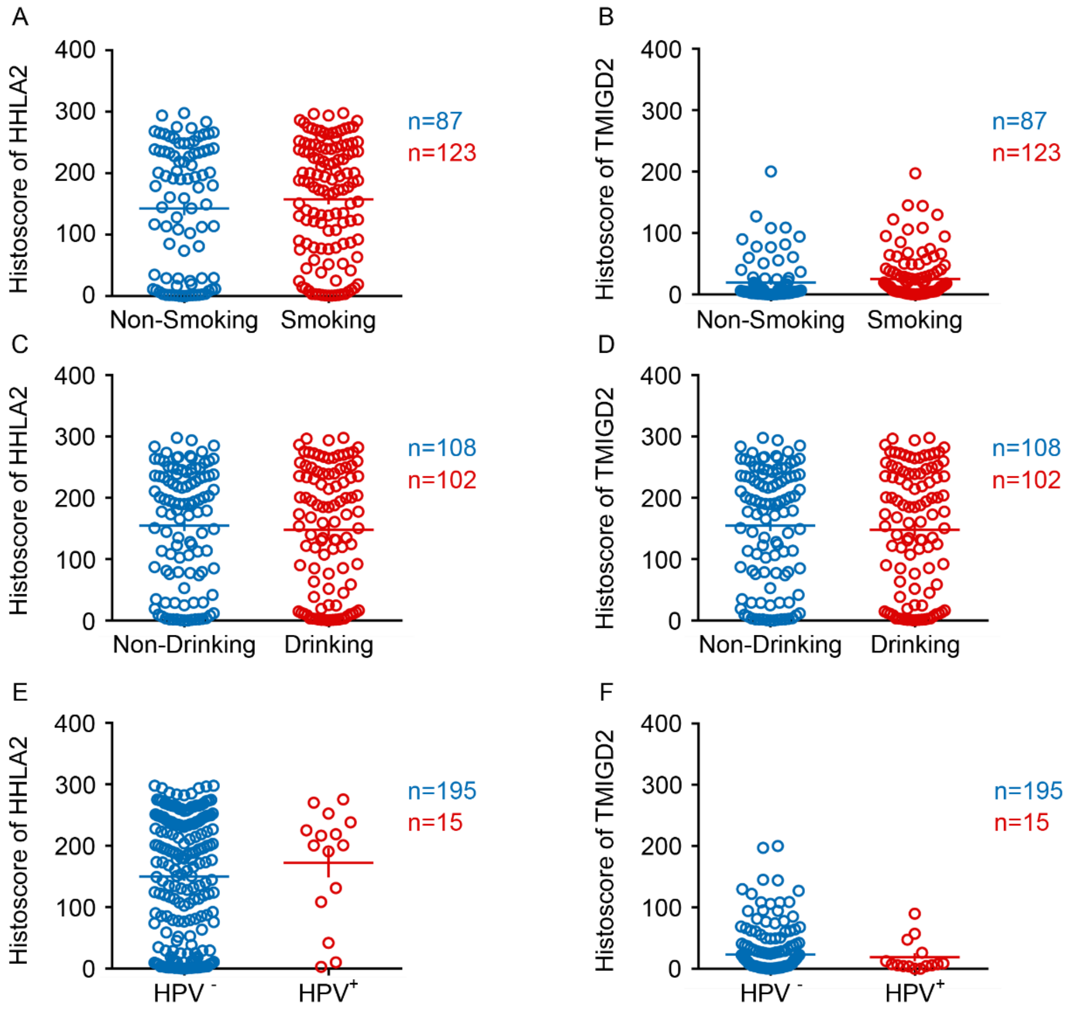

Figure.S2

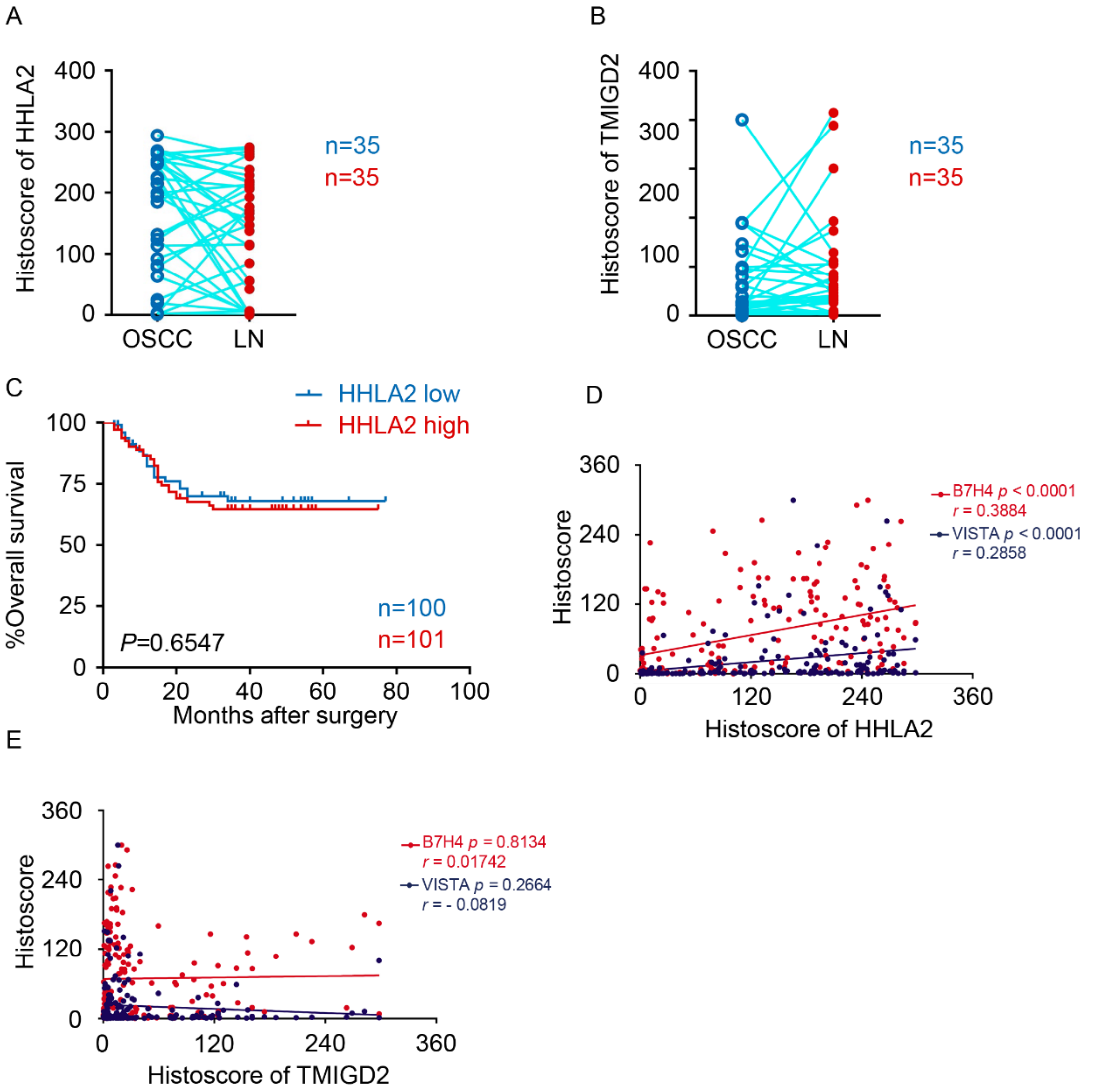
A
Histoscore of HHLA2
400
300
200
100
0
OSCC
LN
n=35
n=35
B
Histoscore of TMIGD2
400
300
200
100
0
OSCC
LN
n=35
n=35
C
HHLA2 low
HHLA2 high
%Overall survival
100
75
50
25
0
P=0.6547
n=100
n=101
0
20
40
60
80
100
Months after surgery
D
Histoscore
360
240
120
0
B7H4 p < 0.0001
r = 0.3884
VISTA p < 0.0001
r = 0.2858
0
120
240
360
Histoscore of HHLA2
E
Histoscore
360
240
120
0
B7H4 p = 0.8134
r = 0.01742
VISTA p = 0.2664
r = - 0.0819
0
120
240
360
Histoscore of TMIGD2

Supplementary figure captions

Figure.S1 HHLA2 and TMIGD2 expression level independent with clinical parameters. **A.** There was no significant difference of HHLA2 expression level was observed between smoking group and non-smoking group. **B.** There was no significant difference of TMIGD2 expression level was observed between smoking group and non-smoking group. **C.** There was no significant difference of HHLA2 expression level was observed between drinking group and non- drinking group. **D.** There was no significant difference of TMIGD2 expression level was observed between drinking group and non-drinking group. **E.** There was no significant difference of HHLA2 expression level was observed between HPV-negative group and HPV-positive group. **F.** There was no significant difference of TMIGD2 expression level was observed between HPV-negative group and HPV-positive group.

Figure.S2 **A.** There was no significant difference of HHLA2 expression level was observed between metastatic lymph node and its' corresponding OSCC. **B.** There was no significant difference of TMIGD2 expression level was observed between metastatic lymph node and its' corresponding OSCC. **C.** The overall survival rate of the group of patients with lower HHLA2 expression significantly different with the group of patients with higher HHLA2 expression (n = 101, $p = 0.0314$, the median expression of HHLA2 was used as the cut-off).

Supplementary Table 1

HHLA2 overexpression could not independently predict prognosis at median cutoff

Parameters	HR (95%CI)	*P* value
Gender	0.903 (0.405-2.009)	0.802
Age	1.804 (0.978-3.328)	0.059*
Pathological grade		
II vs. I	20.965 (2.815-159.154)	0.003*
III vs. I	13.338 (1.690-105.251)	0.014*
Tumor size		
T2 vs. T1	1.112 (0.437-2.833)	0.823
T3 vs. T1	1.792 (0.655-4.903)	0.256
T4 vs. T1	2.004 (0.671-5.991)	0.213
Node stage		
N1+N2 vs. N0	1.194 (0.665-2.142)	0.553
HHLA2	1.115 (0.623-1.996)	0.713

Cox proportional hazards regression model

HR hazard ration, *95% CI* 95% confidence interval

*$p < 0.05$

Supplementary Table 2

HHLA2 overexpression could not independently predict prognosis at best cutoff

Parameters	HR (95%CI)	*P* value
Gender	1.009 (0.449-2.266)	0.982
Age	1.878 (1.016-3.470)	0.044*
Pathological grade		
II vs. I	21.333 (2.860-159.115)	0.003*
III vs. I	13.447 (1.706-106.014)	0.014*
Tumor size		
T2 vs. T1	1.233 (0.448-3.116)	0.659
T3 vs. T1	2.059 (0.753-5.627)	0.159
T4 vs. T1	2.110 (0.706-6.312)	0.181
Node stage		
N1+N2 vs. N0	1.083 (0.598-1.960)	0.793
HHLA2	1.941 (0.908-4.151)	0.087

Cox proportional hazards regression model

HR hazard ration, *95% CI* 95% confidence interval

*$p < 0.05$
